# Serological Comparison of Native Antigen ELISAs with Rapid ICT Test Kits for the Diagnosis of Human Alveolar and Cystic Echinococcosis in China

**DOI:** 10.3390/tropicalmed9020044

**Published:** 2024-02-07

**Authors:** Shu-Kun Yang, Wei Zhang, Na Zhu, Donald P. McManus, Darren J. Gray, Archie C. A. Clements, Angela M. Cadavid Restrepo, Gail M. Williams, Ting Zhang, Guo-Rong Ma, Yan-Hui Yang, Yu-Rong Yang

**Affiliations:** 1 Department of Radiology, The Second Affiliated Hospital of Ningxia Medical University, The First People’s Hospital of Yinchuan City, Yinchuan 750001, China; shukunyang@163.com; 2 Department of Pathogenic Biology & Medical Immunology, School of Basic Medical Science, Ningxia Medical University, Yinchuan 750004, China; zhangwei@nxmu.edu.cn (W.Z.); 225344247@nxmu.com (N.Z.); guorongma8000@163.com (G.-R.M.); yyhysf@163.com (Y.-H.Y.); 3 Molecular Parasitology Laboratory, Global Health & Tropical Medicine, QIMR Berghofer Medical Research Institute, Brisbane, QLD 4006, Australiadarren.gray@qimrberghofer.edu.au (D.J.G.); 4 Department of Global Health, Research School of Population Health, Australian National University, Acton, ACT 2600, Australia; a.clements@qub.ac.uk; 5 Infectious Disease Epidemiology Unit, School of Public Health, University of Queensland, Brisbane, QLD 4006, Australia; a.cadavidrestrepo@sph.uq.edu.au (A.M.C.R.); gail.williams@sph.uq.edu.au (G.M.W.); 6 Faculty of Health Sciences, Curtin University, Perth, WA 6102, Australia; 7 NHC Key Laboratory of Parasite and Vector Biology, National Institute of Parasitic Diseases, Chinese Center for Disease Control and Prevention (Chinese Center for Tropical Diseases Research), Shanghai 200025, China

**Keywords:** alveolar echinococcosis, cystic echinococcosis, egg exposure of *Echinococcus granulosus* and *E. multilocularis*, serological diagnosis, native antigen ELISAs, ADAMU-AE/CE kits

## Abstract

Background: The aim of this study was to compare the diagnostic performance of native antigen ELISAs and ADAMU-AE/CE commercial ICT test kits in subjects either exposed to *Echinococcus* infection or with clinically diagnosed alveolar (AE) or cystic (CE) echinococcosis. Methods: A total of 370 subjects with a previous clinical confirmation of CE or AE from northwestern China were recruited. Serum samples were also obtained from 3923 children/teenagers during a community survey. All sera were tested using native antigen ELISAs. The ADAMU-AE/CE test kits were subsequently used for the serology of the 370 clinically confirmed individuals and of 251 children/teenagers that were ELISA antibody-positive for both *Echinococcus* species but ultrasound-negative during baseline survey. An analysis of the association between the serological tests and ultrasound classification was carried out amongst 89 AE and 164 CE cases. A Kappa consistency analysis was undertaken to compare the diagnostic performance of the native antigen ELISAs and the ADAMU kits and the ultrasound imaging results. The χ² test was also used for a comparison of the different seropositivity rates between the groups. Findings: There was poor consistency (Kappa = 0.26 and 0.28 for AE and CE respectively) between the native antigen ELISAs and the ADAMU kits for the diagnosis of AE and CE among the cases and the surveyed children/teenagers, but a relatively good consistency (Kappa = 0.63) between the ADAMU-AE kit and ultrasound observations for the AE cases. Additionally, of the 251 teenagers co-positive for both AE and CE antibodies by the native antigen ELISAs, only one was found positive by the ADAMU-AE kit, verified as a new AE case on subsequent ultrasound follow-up. The remainder (N = 250) were negative by serology using the ADAMU-AE/CE kits and by ultrasound examination. The two native antigen ELISAs did not discriminate well between cases of clinically diagnosed AE and CE. In contrast, ADAMU-AE and ADAMU-CE commercial ICT test kits readily differentiated cases of AE from CE with specificities of 99% for AE and 100% for CE. Conclusions: The ADAMU-AE/CE kits proved reliable, accurate, and amenable diagnostic tools in the clinical setting for confirmation of suspected AE/CE cases. The native antigen ELISAs tests can provide useful information on the level of human exposure to *Echinococcus* infection.

## 1. Introduction

Echinococcosis (hydatid disease) is a chronic zoonosis that is endemic in many countries worldwide. *Echinococcus granulosus* and *E. multilocularis*, which cause cystic and alveolar echinococcosis (CE and AE), respectively, are the two major species infecting humans [1]. CE and AE cysts grow slowly, and patients in the early stages of disease are generally asymptomatic [1]. The diagnosis of AE and CE by imaging techniques (ultrasound, computed tomography, and magnetic resonance imaging) can reveal space-occupying lesions and their location and size in affected organs, but they cannot always determine the species identity, particularly if the cystic lesions are small in size [2]. Consequently, an effective complementary method is required for the accurate diagnosis of echinococcosis [1,3].

A number of molecular and immunological-based diagnostic approaches have been developed to provide an adjunct for definitive diagnosis with varying success [4]. Serology has proved a useful adjunct to diagnosis and various techniques have been applied [4,5]. Serum antibody measurement, notably using enzyme-linked immunosorbent assays (ELISAs) or Western blotting, can provide up to 95% sensitivity using native antigen extracts or recombinant antigens [5]. However, test specificity can be affected by immunological cross reactivity with antigens found in other helminthiases [5], different types of cancers and liver cirrhosis, or by the presence of anti-P1 antibodies [1,5].

Serological tests using crude native antigens can determine whether a patient has echinococcosis, but the differentiation of AE from CE can be problematic [5,6]. This is an important clinical consideration because the two diseases have different pathological outcomes and treatment and management options [1,2]. Currently, laboratory-based AE sero-diagnosis involves enzyme-linked immunosorbent assay (ELISA)-based tests using recombinant Em18 (rEm18-ELISA) or rEm18 plus the native Em2 antigen purified from *E. multilocularis* larvae (Em2-Em18-ELISA) (BordierAffinity, Crissier, Switzerland), with Western blotting incorporating a whole *E. multilocularis* larval antigen as confirmation for species-specific diagnosis [6]. Current serology for human CE mainly tests for IgG against native or recombinant antigen B by ELISA or Western blotting, with diagnostic performance being dependent on cyst stage, size, and location [7]. An enzyme immunochromatography test (ICT) using the rEm18 antigen has been developed, providing a sensitivity of 94% and a specificity of 95.4% with AE sera [8]. The reliability of the test, currently available as a commercial kit (ADAMU-AE kit, ICST Co. Ltd., Saitama, Japan), was recently verified as a simple, reliable, and easy-to-use tool for the clinical diagnosis of AE cases using a panel of French sera [6]. A similar ICT test (ADAMU-CE), incorporating recombinant antigen B, is also commercially available from the same company and has been tested effectively using the sera of patients with CE from hyper-endemic areas [7].

An antibody response to *E. granulosus* or *E. multilocularis*, detectable by serological methods, can occur for the following reasons: (I) as a result of a new infection through the ingestion of eggs and the hatching of oncospheres that penetrate the intestinal mucosa and release antigens [1,9]; or (II) by an established infection whereby larval antigens are released from established cystic or alveolar lesions [2,9]. The current study aimed to evaluate the value of serology for the specific diagnosis of new and established infections of *E. granulosus* and *E. multilocularis* using panels of Chinese sera, native antigen ELISAs and the ADAMU-AE and ADAMU-CE commercial ICT test kits, which incorporate recombinant antigens.

## 2. Materials and Methods

### 2.1. Ethical Approval

This study was approved by the Ethics Committee of Ningxia Medical University (ref. number 2014-039) and the QIMR Berghofer Medical Research Institute Human Research Ethics Committee (ref. number P1366).

Methods were carried out in accordance with relevant guidelines and regulations and informed consent was obtained from all subjects and/or their legal guardian(s).

### 2.2. Study Subjects

Two subject groups from northwest China were recruited for the study comprising the following: (1) individuals with a clinical history of echinococcosis; (2) children and teenagers (6–18 years) residing in an area highly endemic for echinococcosis in Ningxia, China.

Written consent was received from all adult participants after an explanation of the study purposes and any possible risk when the CE/AE patients (adult participants) attended the periodic follow-up visits at clinics of local Centers of Disease Control and Prevention (CDC) in different provinces. The consent of children participants was obtained from parents or guardians on behalf of their children with a written consent letter after obtaining the child participant’s oral agreement when the survey was undertaken in Ningxia. The patients’ data were collected from local CDCs and the children/teenagers’ data were obtained from community survey. All the data were analysed after being anonymised.

#### 2.2.1. Individuals with a Clinical History of Echinococcosis

As a routine procedure of the Chinese Central Health Authority, individuals registered by the Centres of Disease Control and Prevention (CDC) with a previous surgical history or clinical confirmation of CE or AE are periodically followed up with to determine their current disease state. For the current study, 370 subjects from echinococcosis-endemic regions of northwestern China were recruited when they attended their routine clinical follow-up. Demographic and clinical information, including ultrasound and a 5 mL blood sample, were collected; sera were separated from the blood samples and stored at −80 °C until use.

Based on the ultrasound observations, CE patients were classified into five groups (CE1–CE5) according to the WHO informal working group on echinococcosis [1,5,9]. Due to the limited numbers of patients with either CE4 or CE5, these categories were combined. For AE, classification was based on ultrasound descriptions as infiltrative, with liquefaction, and/or with calcification (rather than on the WHO-endorsed IWGE PNM system, which requires high-resolution imaging that was not available in the current study). Additionally, if AE and CE lesions were both found in an individual, the patient was diagnosed as being co-infected [10]. Due to the follow-up of the registered patients from rural endemic areas, many patients’ records in this study showed a treatment history of near 10 or over 10 years. Among those patients, 51% had AE with scatted calcified lesions and 20% had CE with dead cysts.

#### 2.2.2. Community Survey of Children and Teenagers

A community survey investigating the risk factors for *Echinococcus* spp. transmission was undertaken. The survey comprised a questionnaire, abdominal ultrasound, and sampling of blood (3–5 mL) amongst 3923 children/teenagers in the Xiji and Guyuan counties, Ningxia Hui Autonomous Region (NHAR), which are both known to be co-endemic for *E. multilocularis* and *E. granulosus*. Sampled sera were stored at −80 °C as described above until use. All individuals were recorded as ultrasound-negative during the baseline survey.

### 2.3. Serological Tests

**Figure 1:**
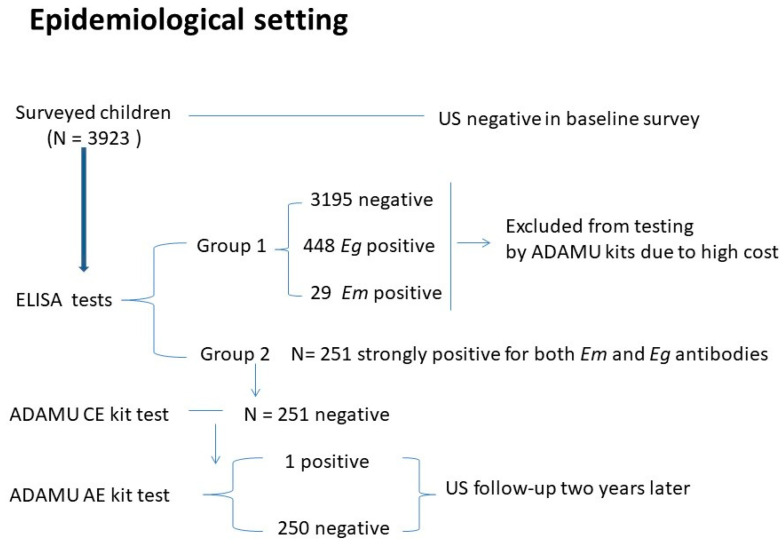
Epidemiological setting: a baseline survey by abdominal ultrasound and serology was undertaken amongst children and teenagers (aged 6–18 years) in an area endemic for echinococcosis, Ningxia, China. A follow-up (mainly involving abdominal ultrasound and chest X-ray) amongst (Group 1 children) the subjects who were serum antibody-positive for both *E. granulosus* and *E. multilocularis* was undertaken two years later.

Detection of either anti-*E. multilocularis* or anti-*E. granulosus* antibodies in serum was conducted using two approaches: (1) validated fluid (HCF) or a crude extract of *E. multilocularis* tissue/protoscoleces (*Em*P) [5,11,12]; and (2) commercial ICT kits (ADAMU-AE/CE kits, ADTEC Co., LTD. Japan) with recombinant/affinity purified antigen B1 (rAgB1) for CE diagnosis [7] and recombinant/affinity purified *E. multilocularis* (rEm18) antigen for AE diagnosis [6]. For the survey involving children and teenagers, testing was performed sequentially. Sera that were positive for both *Echinococcus* species (co-positive antibodies against *E. multilocularis* and *E. granulosus* antigens) according to native antigen ELISA were further tested using the ADAMU-AE and CE kits. Any individuals who were serologically co-positive for AE and CE by ELISA using these kits were then followed up with by ultrasonography and questionnaire (Figure 1).

#### 2.3.1. Native Antigen ELISAs

Microtiter plates with 96 wells (Lot28813025, Corning Incorporated, Oneonta, NY, USA) were pre-coated with 100 µL per well of native *E. granulosus* (HCF) or *E. multilocularis* (*EmP*) antigens (1:1000 dilution) and incubated at 4 °C overnight and then washed three times (3 min each time; tapped dry) with 0.15 M phosphate buffered saline (PBS) in pH 7.2 containing 0.1% Tween-20 (PBST). The plates were then blocked (100 µL per well) with 0.3% PBST containing 5% (*w*/*v*) foetal bovine serum (Lot 111210, Technology Co Ltd., Hangzhou of Zhejiang, China) (PBST-F) at RT for 1 h, then they were rinsed three times with 0.1% PBST. Each plate included positive and negative control serum samples and two wells without antigen to act as blank controls. Each plate was loaded with 100 µL of diluted serum (1:50 dilution in PBST-F) per well and incubated for 40 min at RT. The wells were rinsed three times with 0.1% PBST before applying 100 µL of anti-human peroxidase-conjugated IgG-Fc antibodies (mouse monoclonal H2 with HRP, Abcam, CA, USA) at a 1:10,000 dilution at RT for 30 min. The plates were again rinsed three times with 0.1% PBST and tapped dry, then 100 µL of tetramethyl benzidine (TMB) substrate in 3% PBST were added per well, and the plate was left for 15 min at RT. The reaction was quenched with 50 µL of sulphuric acid (2 M) per well. All wells were read on a BioRAD 680 microplate ELISA reader at 490 nm (BioRAD, Hercules, CA, USA).

Positive cut-off values were determined by the mean values of the optical density (OD) plus three standard deviations of the uninfected negative control sera [13]. A seropositive OD value was assessed as being equal to or greater than the cut-off value.

#### 2.3.2. ADAMU-AE and CE Test Kits

The ADAMU-AE and CE test kits were used according to the instructions provided by ICST Co. Ltd., Saitama, Japan. In brief, the rAgB1 or rEm18 antigens (test line) and anti-goat immunoglobulin G (IgG) (control line) are immobilised onto a nitrocellulose membrane and placed in a plastic test cassette [14] with 5-Bromo-4-chloro-3-indolylphosphate (BCIP) as a substrate for colour development. For the assay, 20 µL of human serum sample and 40 µL of serum dilution buffer containing 0.1 mg/mL alkaline phosphatase-conjugated goat anti-human IgG antibody are mixed in a sample cup and 20 µL of the mixture is then applied onto the sample window of the cassette. Antibodies to AE or CE in the serum bind the conjugate, and the composite bodies bind to rAgB1 or rEm18 which are immobilized in the result window. Then BCIP is hydrolysed by alkaline phosphatase to form a blue line which determines whether antibodies are present or not. A serum sample is considered positive if two blue colour lines are present after 20 min, indicating the presence of the specific anti-rEm18 or anti-rAgB1 antibodies (test line), and if the control line (anti-goat IgG) is visible (indicating that the test was performed correctly). The presence of only one band within the result window indicates a negative result.

### 2.4. Statistical Analysis

A Kappa consistency analysis between the ultrasound outcomes and serology outcomes (the US and the native antigen ELISA test; the US and ADAMU kits; native antigen ELISAs and ADAMU kits) for AE and CE was performed using SPSS 20.0, whereby a Kappa-value > 0.75 was taken to indicate a strong consistency and a Kappa-value < 0.4 was taken to indicate a poor consistency. The sensitivity and specificity of the two serological test systems (ELISA and ADAMU-AE/CE kits) were assessed using the ultrasound outcomes. Chi-square tests (χ^2^ test) were used to compare the seropositive rates between groups. A value of *p* < 0.05 was considered significant.

## 3. Results

### 3.1. Studied Subjects

#### 3.1.1. Clinical Diagnosed Cases

Totals of 269 cases with CE, 95 cases with AE, and 6 patients with co-infection of both AE and CE were diagnosed in patients with ages ranging from 4–78 years (mean of 40 years); there were 134 males and 236 females; 224 were Han, 56 Hui, 65 Mongolian, and 25 Kazak.

#### 3.1.2. Community Survey Individuals

In the children/teenagers group, their ages ranged from 6–18 years (mean = 12 years). A total of 1962 individuals were male, and 1961 were female; 1603 were Han, 2313 were Hui, and 10 subjects had no ethnicity recorded.

### 3.2. Serological Outcomes

#### 3.2.1. Results of Subjects with a Clinical History of Echinococcosis

**Figure 2:**
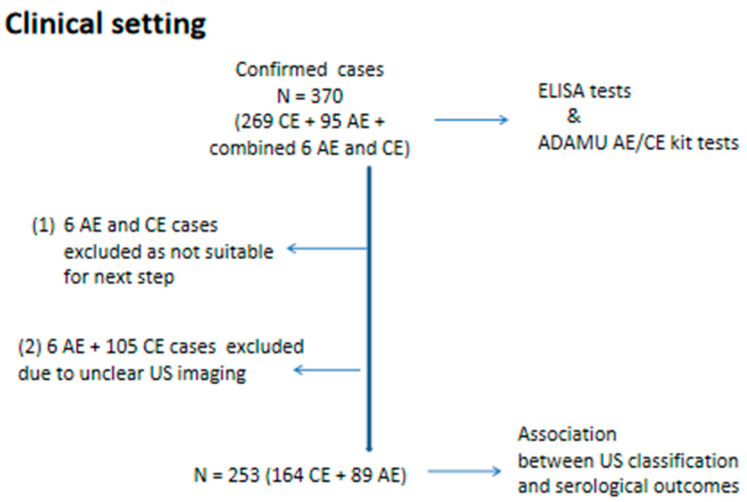
Clinical setting: all recruited AE and CE cases were diagnosed in different hospitals or clinics in different years within two areas (Ningxia and Xinjiang Autonomous Regions) endemic for echinococcosis and were registered at the county level in local Centres of Disease Control for continuous albendazole treatment and regular follow-up.

In total, 370 subjects under investigation had a complete clinical record, including imaging observations or key indicators for positive diagnosis of echinococcosis according to the WHO-IWGE consensus for the diagnosis and treatment of CE and AE in humans [1,15]. Accordingly, serology was undertaken on sera collected from these 370 subjects using the native antigen ELISAs and the ADAMU-AE and CE kits, and the results are shown in Table 1. The serology results for 89 of the 95 AE cases and 164 of the 269 CE cases were used subsequently for assessment of association with the US classification (Figure 2 and Table 2).

#### 3.2.2. Community Survey of Children and Teenagers

On the basis of results obtained from the sera tested for the presence of serum AE/CE antibodies by the native antigen ELISAs for all 3923 surveyed individuals, subjects were placed into two groups. Group 1 included all subjects (N = 251) who were strongly seropositive for both *Em* and *Eg* antibodies (Table 3). Group 2 included individuals who were either seropositive for *Em* antibodies (N = 29) or *Eg* antibodies (N = 448) or who were AE/CE seronegative (N = 3195) (Table 3). The 251 group I sera were further tested using the ADAMU-AE and CE kits (Table 3), but only one serum sample proved positive with the ADAMU-AE kit (Figure 1).

All individuals seropositive by either the native ELISAs or ADAMU kits were subjected to repeat abdominal US and chest X-ray examination at a follow-up two years later. The single AE patient (aged 17 years) who was seropositive by native antigen ELISA and the ADAMU-AE kit, was classified as P1, according to the WHO-IWGE PNM classification system [1,15], but the other 250 individuals were negative by imaging.

#### 3.2.3. Consistency between Imaging Outcomes and the Two Serological Methods

Poor consistency (Kappa = 0.26 and 0.28 for AE and CE, respectively) was evident between the results obtained by the two different assays (native antigen ELISAs and ADAMU kits) for the diagnosis of AE and CE patients. When comparing the US results with the two serology methods, there was a relatively good level of consistency evident between the ADAMU-AE test kit and the US observations for the AE cases (Kappa = 0.63), but there was poor consistency between the US and the native antigen ELISA test (Kappa = 0.08). Amongst the CE cases, there was poor consistency in the comparison both between the US observations and native antigen ELISA (Kappa = 0.09) and between the US and the ADAMU-CE test kit (Kappa = 0.09).

In the community survey of children/teenagers, as indicated earlier, apart from one individual who was US-positive and positive using the ADAMU-AE kit, all the other individuals in Group I that were US-negative were also negative by either the ADAMU-AE or -CE kits. This emphasized the inconsistency between the ICT- and native antigen ELISA-based tests with the latter showing a proportion (251/3923) that was strongly seropositive for both *E. multilocularis* and *E. granulosus* antibodies (Table 3).

#### 3.2.4. The Sensitivity/Specificity and Level of Cross-Reactivity of the Two Serological Methods

Based on the positive diagnosis by imaging (mainly US), the sensitivity (33%) of the native AE antigen ELISA was significantly lower (*p* < 0.05) than the ADAMU-AE kit (71%) (Table 4). In contrast, the sensitivity of the native CE antigen ELISA (77%) was significantly higher (*p* < 0.05) than the ADAMU-CE kit (46%) (Table 4).

The specificities of the native AE antigen ELISA (93%) and native CE antigen ELISA (82%) were significantly lower (*p* < 0.05) than the ADAMU-AE kit (99%) and the ADAMU-CE kit (100%), respectively (Table 4).

The cross-reactivity rate in the detection of AE cases with the native CE antigen-ELISA (68%) was significantly higher (*p* < 0.05) than with the ADAMU CE kit (34%). The cross-reactivity rate in the detection of CE cases with the native AE antigen ELISA was 24% compared with 7% by the ADAMU AE kit (*p* < 0.05), indicating that cross-reactivity was more frequent when using AE patient serum than CE patient serum and that the ADAMU kits, particularly the ADAMU-AE kit, produced lower levels of cross-reactivity than the native antigen ELISAs (Table 4).

#### 3.2.5. Association between CE and AE Imaging Classification and the Two Serological Results

The highest level of seropositivity by the native CE antigen ELISA was evident in CE patients with CE2-type cysts, and this was significantly higher (*p* < 0.05) than in the three other classified groups (Table 2). There was also a significant difference in antibody-positive results (*p* < 0.05) between the CE1 and CE4/CE5 types but not between the CE1 and CE3 types or between the CE3 and CE4/CE5 groups (Table 2). There were no significant differences in antibody-positive cases (*p* > 0.05) among the four groups tested using the ADAMU-CE kit.

The antibody positivity rate determined for the AE cases was substantially lower using the native AE antigen ELISA than that obtained with the ADAMU-AE kit (Table 2). There was no difference in antibody positivity rate between the three classified types of lesions (infiltrating, liquefied, calcified) determined by the native AE antigen ELISA. The calcified lesion group had the lowest antibody positivity rate determined using the ADAMU-AE kit compared with the other two groups (*p* < 0.05) (Table 2).

## 4. Discussion

The aim of this study was to compare the diagnostic performance of validated native *Echinococcus* antigen ELISA tests with commercial ICT-based (ADAMU-CE/AE) kits by assessing their value in defined clinical and epidemiological settings. Human echinococcosis is acquired through the oral ingestion of viable parasite eggs contaminating the environment [11,16]. The accurate diagnosis of individuals exposed to *Echinococcus* eggs (egg antigen stimulation) and those with clinical manifestations (larval antigen stimulation) are important considerations for effective echinococcosis control programs [17,18]. The identification of infected subjects is usually based on evidence from symptomatic clinical findings, epidemiological data, and the combined use of imaging techniques, particularly US, and serology [1,2,15].

US is useful for assessing the presence and staging of a cystic or alveolar lesion [3,9] and for differentiating echinococcosis from other liver-occupying lesions such as cancer [19], but AE and CE serology is a valuable adjunct for correct clinical diagnosis and the epidemiological surveillance of high-risk populations [20]. Positive AE/CE serology can be correlated with the progressive development of a lesion [15], an aborted lesion, spontaneous cure without any apparent lesion, or an early infection before any lesion is detectable by imaging [1,2]. Therefore, whereas accurate imaging and clinical information are essential to reveal AE/CE cases which would benefit from treatment [9,15], serology can provide useful information on the infection pressure of a particular area due to the presence of echinococcal eggs contaminating the local environment [11,16].

*E. granulosus* is generally not invasive after human infection, growing as a clearly demarcated space-occupying lesion. Nevertheless, the spread of the parasite may occur after the spontaneous, traumatic, or iatrogenic rupture of cysts which can result in complications including abdominal pain, secondary bacterial infection, allergic reactions, anaphylaxis, and death [1,15]. In contrast, however, *E. multilocularis* behaves in a way similar to a malignant tumour, showing both local invasive and metastatic growth [21]. Although the growth rate is much slower than in neoplasia, AE is a serious disease that can result in a death rate of up to 90% ten years post-disease onset if left untreated [22]. Early diagnosis of AE reduces the need for radical treatment such as major surgery and results in fewer unresectable lesions [1,2,15]. Accordingly, these different growth and disease patterns require the early, accurate, and specific diagnostic differentiation of the two *Echinococcus* spp. so that appropriate treatment can be commenced to improve prognosis. The early clinical diagnosis of both AE and CE is difficult, since both diseases are typically asymptomatic long term [15], but early detection is important as it can result in earlier and more effective chemotherapeutic treatment [2,15,22]. The early detection of human infections of *E. multilocularis* and *E. granulosus* is also important in echinococcosis surveillance and in monitoring control programs [9,11,17].

Several diagnostic assay platforms, principally ELISA and Western blotting, for AE and CE serology testing for IgG antibodies have been developed incorporating various antigen preparations (e.g., native antigens from *E. granulosus* hydatid cyst fluid (HCF) and native or recombinant antigen B for CE [23,24]; native *E. multilocularis* tissue/protoscoleces (*Em*P), native or recombinant rEm18, and native Em2-Em18 for AE) with variable levels of sensitivity, specificity, and cross-reactivity [25]. The two native antigen ELISAs used in our study did not discriminate well between cases of diagnostically proven AE and CE. In contrast, the ADAMU-AE and ADAMU-CE commercial ICT test kits using the recombinant Em 18 and Eg B8-1 antigens, respectively, readily differentiated cases of AE from CE, with a specificity of 99% for AE and 100% for CE, thereby providing reliable, accurate, and simple diagnosis in the clinical setting.

We were also able to correlate the serological profiles of patients with different stages of CE or AE determined by imaging. We observed the highest rates of native antigen ELISA-seropositivity in CE patients with active disease (types CE1 and CE2) and the lowest in patients with inactive disease (types CE4 and CE5), reflecting the strong host immune response generated during the early stages of *E. granulosus* cystic development (active disease) and a weak or undetectable host immune response in the later inactive stages of CE in accordance with the US classification [1,15,24]. Whilst the ADAMU-CE kit was unable to discriminate between the different CE cyst types, the native antigen ELISA was able to detect a significantly higher percentage of the active/early stages (types CE1 and CE2) than the ADAMU-CE kit, although similar antibody-positive rates were determined by the two detection methods for the late stages (types CE3 and CE4/CE5).

In direct contrast to the serological findings with the CE patients, there was a significantly higher level of seropositivity evident in AE patients using the ADAMU-AE kit compared with the native antigen ELISA. The ADAMU kit also showed that higher antibody-positive rates occurred in AE patients with infiltrating and liquefied lesions compared with those having calcified cysts.

During the course of this study, a number of patients were recruited with dead/inactive AE or CE cystic lesions due to them being subjected to long term (>10 years for some subjects) albendazole treatment. The inclusion of these patients (51% AE patients with calcified lesions and 20% CE patients with dead cysts) would likely have decreased the sensitivity of the ADAMU-AE/CE ICT test kits. Another issue was the relative lack of species-specificity of the AE and CE native antigen ELISA tests, as serological cross-reactivity was commonly observed [13]. Additionally, the correlative analysis of disease courses or ultrasound classifications with both serological methods presented uncoordinatedly in either AE or CE, which may be explained by the limited number of cases of either kind of patient. Overall, the ADAMU-CE kit exhibited a higher level of specificity than the ADAMU-AE kit, although both ICT kits were far superior to the native antigen ELISAs for the confirmation of subjects with AE or CE.

## 5. Conclusions

In conclusion, this study evaluated and compared the efficacy of two commercial rapid ICT AE and CE test kits with ELISA-based methods incorporating crude or partially purified *E. granulosus* and *E. multilocularis* metacestode-derived native antigens as tools in a sero-epidemiological study of human AE and CE prevalence and in the clinical differential diagnosis of patients suspected as having AE or CE. The ADAMU-AE and CE kits proved more effective in the clinical setting for the confirmation of suspected AE/CE cases, whereas the native antigen ELISA tests can provide useful information on the environmental rates of *Echinococcus* egg exposure at the population level.

## Figures and Tables

**Table 1:** Results of serology using native antigen ELISAs and ADAMU kits in patients with a history of alveolar (AE) and/or cystic (CE) echinococcosis.

US-Positive Patients	Number Tested	ELISA				ADAMU kit			
		Em	Eg	Em/Eg	No Ab	Em	Eg	Em/Eg	No Ab
AE	95	2	36	29	28	39	4	28	24
CE	269	2	144	63	60	6	109	14	140
AE and CE	6	0	4	1	1	0	1	2	3
Total	370	4	184	93	89	45	114	44	167

Eg, *Eg* antibody-positive; Em, *Em* antibody-positive; Em and Eg, *Em* and *Eg* antibody-positive; No Ab, antibody-negative.

**Table 2:** Correlation of AE/CE patients’ imaging classification with seropositivity by native antigen ELISAs and ADAMU test kits.

	ELISA		ADAMU Kit	
US Classification	Po./All (%)	*p*-Value	Po./All(%)	*p*-Value
CE types				
CE1 ^a^	20/30 (67)	<0.01 ^a,b/a,d^	11/30 (37)	>0.05 (df = 3)
CE2 ^b^	68/78 (87)	<0.01 ^b,c/b,d^	39/78 (50)	
CE3 ^c^	14/23 (61)	>0.05 ^a,c^	13/23 (57)	
CE4/CE5 ^d^	11/33 (33)	>0.05 ^c,d^	10/33 (30)	
AE types				
Infiltrating ^1^	10/29 (34)	>0.05 (df = 2)	25/29 (86)	1.0 ^1,2^
Liquefied ^2^	4/15 (27)		13/15 (87)	<0.05 ^2,3^
Calcified ^3^	14/45 (31)		24/45 (53)	<0.01 ^1,3^

Po., seropositive individuals; all, all tested individuals; df, degrees of freedom. In CE types, the labels of ^a^, ^b^, ^c^, ^d^ represented each group of CE1, CE2, etc., since the single letters can be easy to facilitate the marking of the compared groups in the *p*-value column. The same reason, for ease of marking, in AE types, the labels of ^1^, ^2^, ^3^ represented each group of Infiltrating AE, Liquefied AE, Calcified AE, respectively.

**Table 3:** Results of serology using native antigen ELISAs in the community-surveyed children/teenagers and by ADAMU-AE/CE kits in a proportion (Group 1) of surveyed individuals.

US-Negative	Number Tested	ELISA				ADAMU Kit			
Subjects		Em	Eg	Em/Eg	No Ab	Em	Eg	Em/Eg	No Ab
Group 1	251	/	/	251	/	1	0	0	250
Group 2	3672	29	448	/	3195	N/A	N/A	N/A	N/A
Total	3923	29	448	251	3195	1	0	0	250

Group 1 comprises individuals strongly positive for both *Em* and *Eg* antibodies by native antigen-ELISA. Group 2 comprises individuals either positive for *Em* or for *Eg* antibodies or antibody-negative for both species by native antigen ELISA; N/A, the test was not carried out.

**Table 4:** The sensitivity, specificity, and level of cross-reactivity of the native antigen ELISAs compared with the ADAMU test kits for AE and CE diagnosis.

Category	AE			CE		
	ELISA	ADAMU Kit	*p*-Value	ELISA	ADAMU Kit	*p*-Value
Sensitivity	31/95 (33%)	67/95 (71%)	<0.05	207/269 (77%)	123/269 (46%)	<0.05
Specificity	3643/3923 (93%)	250/251 (99%)	<0.05	3224/3923 (82%)	251/251 (100%)	<0.05
Cross-reactivity	65/95 (68%)	32/95 (34%)	<0.05	65/269 (24%)	20/269 (7%)	<0.05

## Data Availability

Data are available upon request.
